# Porcine Circoviruses and Xenotransplantation

**DOI:** 10.3390/v9040083

**Published:** 2017-04-20

**Authors:** Joachim Denner, Annette Mankertz

**Affiliations:** Robert Koch Institute, Nordufer 20, 13353 Berlin, Germany; MankertzA@rki.de

**Keywords:** porcine circoviruses, transspecies transmission of viruses, xenotransplantation, virus safety of xenotransplantation

## Abstract

Allotransplantation and xenotransplantation may be associated with the transmission of pathogens from the donor to the recipient. Whereas in the case of allotransplantation the transmitted microorganisms and their pathogenic effect are well characterized, the possible influence of porcine microorganisms on humans is mostly unknown. Porcine circoviruses (PCVs) are common in pig breeds and they belong to porcine microorganisms that still have not been fully addressed in terms of evaluating the potential risk of xenotransplantation using pig cells, tissues, and organs. Two types of PCVs are known: porcine circovirus (PCV) 1 and PCV2. Whereas PCV1 is apathogenic in pigs, PCV2 may induce severe pig diseases. Although most pigs are subclinically infected, we do not know whether this infection impairs pig transplant functionality, particularly because PCV2 is immunosuppressive. In addition, vaccination against PCV2 is able to prevent diseases, but in most cases not transmission of the virus. Therefore, PCV2 has to be eliminated to obtain xenotransplants from uninfected healthy animals. Although there is evidence that PCV2 does not infect—at least immunocompetent—humans, animals should be screened using sensitive methods to ensure virus elimination by selection, Cesarean delivery, vaccination, or embryo transfer.

## 1. Introduction

Allotransplantation can be associated with transmission of microorganisms which induce severe diseases in the recipient [1,2]. Among the transmitted microorganisms are bacteria [3] and viruses such as the human immunodeficiency virus-1 [4], rabies virus [5,6,7], and human cytomegalovirus (HCMV) [8]. Infection with HCMV is a common complication after transplantation of different organs and contributes significantly to morbidity and mortality, both by direct and indirect mechanisms [9]. Therefore, HCMV status has to be determined and transplantations from HCMV-positive individuals to HCMV-negative individuals are generally avoided (for review see [8,9]). If necessary, an antiviral treatment is available and new antiviral drugs are under development [10]. Xenotransplantation using pig cells, tissues, and organs may also be associated with transmission of microorganisms, including bacteria, viruses, and others from the donor pig [11]. Transmission of porcine cytomegalovirus (PCMV) with the transplant and its increased replication, also called reactivation, on the background of the absence of the pig immune system and of the applied immunosuppression in the non-human primate recipient, was observed after pig kidney transplantations into baboons [12] or cynomolgus monkeys [13]. Transmission of PCMV was also observed after pig heart transplantations into baboons [14]. Although the virus titre in the recipients increases, it is unclear whether PCMV is able to infect cells of the recipient or is replicating only in the cells of the transplant.

There are obvious differences between the transmission inside the human species during allotransplantation and transspecies transmission into a new species during xenotransplantation. Human microorganisms are adapted to humans and can be easily transmitted [1,2,3,4,8,9]. The porcine microorganisms are not adapted to humans, and it is clear that many of them cannot infect human cells due to the absence of a receptor or due to cellular factors restricting replication in human cells. In contrast to human pathogens, sensitive detection methods for porcine microorganisms were developed only in a few specialized laboratories, and it is unclear whether commercial test laboratories can detect low virus load infections, as shown in one case of PCMV infection [15]. Sending identical virus dilutions to different laboratories worldwide for testing, so called round robin tests, may answer this question. The results of the testing will indicate the sensitivity of their methods. In this context, in a recent round robin or ring test including 11 North American laboratories, the most sensitive assay detected DNA levels of a porcine virus about 100,000 times lower than the least sensitive assay [16]. This study demonstrated that the polymerase chain reaction (PCR) assays available in these diagnostic labs vary considerably in their detection limits and quantification.

Even if porcine microorganisms can infect humans and replicate, it is still unclear whether they are pathogenic. For example, hepatitis E virus (HEV) genotype 3 coming from pigs mostly induces diseases in chronically ill and immunosuppressed humans, but not in healthy individuals [17], although the influence of the subclinical infection on the health of the infected person is still unknown.

**Figure 1:**
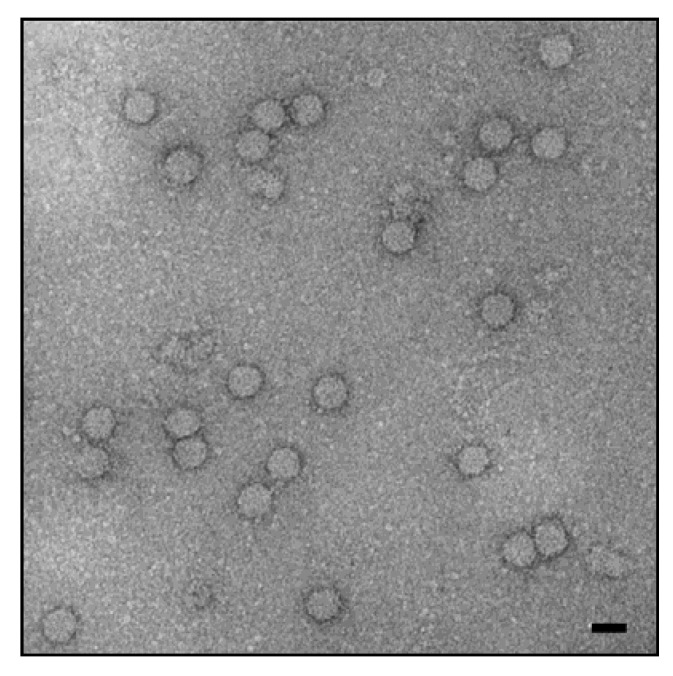
Electron microscopy of porcine circovirus (PCV), negative staining with uranyl acetate. The scale bar corresponds to 20 nm. Hans Gelderblom, Michael Laue, Robert Koch-Institute.

The porcine circoviruses (PCVs) belong to the genus *Circovirus* of the family *Circoviridae* [18]. Porcine circovirus 1 (PCV1) was first described at the Robert Koch Institute, Berlin [19]. Other members of this family are PCV2, several avian circoviruses [18], and recently new circoviruses have been isolated from mammals: bat [20,21], dogs [22,23,24,25], mink [26,27], and others. Circoviruses are non-enveloped spherical (16–18 nm) particles (Figure 1) with a single-stranded and circular small DNA genome. PCVs are the smallest viruses found to be replicating in mammalian cells. PCVs are quite stable, the effectiveness of disinfectants for reducing PCV2 in vitro is variable and PCV2 is very stable in the pig environment. The virus is very resistant under high temperatures and a wide range of pH conditions (for review see [28]). Two major open reading frames (*orfs*) have been recognized. Orf1 encodes the two replicases indispensable for viral replication (Rep and Rep’), and *orf*2 encodes the capsid protein Cap, which is the major structural protein [29]. Three other genes, *orf*3, *orf*4, and *orf*5 encode proteins not essential for virus replication, but involved in the virulence and spread of the virus [30,31]. Cells of the monocyte and macrophage lineage have consistently been shown to be targets for porcine circovirus replication in vivo, and appear to be important in the pathogenesis of the postweaning multisystemic wasting syndrome (PMWS) [32,33,34]. Additionally, a variety of other cell types, including hepatocytes, enterocytes, renal and alveolar epithelial cells, vascular endothelial cells, pancreatic acinar and ductular cells, lymphocytes, smooth muscle cells, and fibroblasts, have also been shown to contain PCV2 antigens and/or nucleic acid [35]. It was shown that heparin, heparan sulphate, and chondroitin sulphate are attachment factors for PCV2 [36], whereas the main receptor is still unknown [37].

PCV1 is apathogenic in pigs, but PCV2 is associated with severe diseases, among them PMWS, which is considered the most significant PCV2-related disease (PCVD) (see Section 3). PCV2 is an immunosuppressive virus, targeting the lymphoid tissues, which leads to lymphoid depletion and immunosuppression in pigs. The virus resides in immune cells, such as macrophage and dendritic cells, and modulates their functions. Upregulation of interleukin (IL)-10 and proinflammatory cytokines in infected pigs may contribute to pathogenesis and co-infection with other pathogens. PCV2 DNA and proteins interact with various cellular genes that control immune responses [38,39,40]. Although numerous reviews summarise the impact of PCV2 on pig production and give detailed descriptions of the pathogenesis of PCV2-induced diseases in pigs [40,41,42,43], this review is the first to analyse the potential impact of PCV2 on xenotransplantation, and to analyse whether PCV2 may represent a risk for xenotransplantation. PCV2 induces severe diseases in pigs, but it remains unclear whether subclinical infections may reduce the quality of the pig transplants, particularly because the virus is immunosuppressive. Vaccination against PCV2 is able to prevent diseases, but in most cases is unable to prevent the transmission of the virus (for details see Section 6). Using sensitive methods will increase the probability of detecting the virus. However, it remains unclear how sensitive these methods should be. At the very least, in order to prevent transmission during xenotransplantation, the sensitivity of the detection methods should allow for the detection of the virus below the load which is able to be transmitted and to induce zoonosis [44]. We also indicate different strategies to eliminate the viruses from the donor pig herd in order to prevent transmission to human recipients.

## 2. Diagnosis and Transmission

PCR is a sensitive method of choice to detect a circovirus infection in viremic animals and different PCR assays, including real-time PCR or quantitative PCR (qPCR) and digital droplet PCR (ddPCR) using specific primers for PCV1 and PCV2 have been developed and applied [45,46,47,48,49,50]. In some cases, PCVs were detected simultaneously with other porcine viruses using multiplex PCR [51,52]. Other detection methods are antibody-based methods such as enzyme-linked immunosorbent assay (ELISA), Western blot analysis, and immunofluorescence [53,54,55,56,57,58]. The use of saliva for antibody detection gained popularity because of the ease of use and associated cost-saving [59]. Serum antibodies to PCV1 and PCV2 have been demonstrated in a large percentage of pigs in different countries at a time when vaccination had not yet been introduced [60]. PCR screening in the year 2000 of randomly collected 109 organ samples from German pigs not affected with PCVD revealed a rate of infection with PCV1 of 5% and with PCV2 of 26.8% [46]. Seroconversion usually occurs by two to four months of age irrespective of whether clinical signs of PCVD are observed. PCV2 is shed for a long time by different routes, both after natural as well as experimental infection [61,62]. Therefore, it easily spreads within the population, mainly by direct contact with contaminated respiratory, digestive, and urinary secretions. Although PCV2 has been identified in the semen of acutely affected boars, transmission of the virus via this route has not been documented in a field setting [63,64].

Based on phylogenetic analysis, PCV2 is divided into different genotypes (PCV2a, PCV2b, PVC2c, PCV2d, PCV2e, PCV2f) [65]. The first three variants show 97%–100% nucleotide identity in the *rep* gene and 91%–96% in the *cap* gene [66]. They are believed to have evolved from a common ancestor 100 years ago [67]. In recent years, evidence has accumulated for a global shift of the main PCV2 genotypes in different countries from PCV2a to PCV2b, which is generally associated with more severe disease symptoms [68,69]. PCV2d was initially identified in Switzerland, now it appears to be widespread in China and North America. During 2012–2013, 37% of all investigated PCV2 sequences from U.S. pigs were classified as PCV2d, and overall data analysis suggests an ongoing genotype shift from PCV2b towards PCV2d [70]. Recombinations and mutations have been often observed and may result in altered fitness or phenotypic properties [71,72,73].

Since changes in the nucleotide sequence of genomic regions used as targets for PCR-detection of PCV may result in false-negative findings, the primers must be checked routinely by a Basic Local Alignment Search Tool (BLAST) search of GenBank for their fitness to detect new variants. If no highly conserved regions can be identified and problems related to genomic variation are anticipated, multiplex PCRs for different viral variants using more than one primer pair or next generation sequencing can be employed.

Recently, a new virus, PCV3, with significant differences in the sequence when compared with PCV1 and PCV2, but more related to a bat-faeces associated circovirus, was described in pigs with cardiac and multi-organ inflammation [74]. Since the pigs were co-infected with other porcine viruses, the pathogenicity of PCV3 warrants further investigations. PCV3 was found to be associated with porcine dermatitis and nephropathy syndrome (PDNS), reproductive failure, and multisystemic inflammation in China [75,76] and in the USA [77]. Sequence analysis showed that the Chinese isolates are the result of a recombination between bat circoviruses [76], and the closest relative of the U.S.A. isolate is a canine circovirus [77].

## 3. PCV2-Related Diseases in Pigs

PCVD was first detected in the early 1990s and has since then emerged as an economically important pig disease worldwide [61]. The main disease induced by PCV2 is PMWS [41,42]. However, PCV2 induces an entire complex of diseases now called PCVD in Europe or PCV-associated disease (PCVAD) in North America [33]. PCVD can be subdivided into PCV2-systemic disease (PCV2-SD, directly replacing PMWS), PCV2-subclinical infection (PCV2-SI), PCV2-reproductive disease (PCV2-RD), and PDNS. PCV2 is necessary but not sufficient for the induction of PCVD. Some purported risk factors include coinfection with other viruses. Porcine reproductive and respiratory syndrome virus (PRRSV) is one of these viruses, it causes the porcine reproductive and respiratory syndrome associated with reproductive failure in breeding stocks and respiratory tract illness in young pigs. Co-infection with porcine parvovirus may also contribute to PCVD as well as nonspecific immune stimulation (e.g., by vaccination).

Clinical signs of the disease include gradual wasting, fever, rough hair coat, dyspnea, pallor, diarrhea, and occasionally icterus. PCVD is characterized by lymphoid depletion, immunosuppression, and inflammation in affected organs. Morbidity varies from 2%–30%, but case fatality is high, approaching 80%. Occasionally, pigs may develop purple skin lesions and nephropathy, likely as an immune mediated sequel to viral infection, termed PDNS [78,79]. Occasionally reproductive failure is observed as abortions, stillbirths, and mummification (PCV2-RD) [62,80,81].

## 4. PCV Does Not Infect Immunocompetent Humans

When trying to infect human cell lines with PCV1 and PCV2, PCV1 persisted in most cell lines without causing any visible changes, while PCV2-transfected cells showed a cytopathogenic effect [82]. Most importantly, in both cases the infection was non-productive [82,83]. Infection with PCV1 was observed in human 293, HeLa, and Chang liver cells, whereas PCV2 infected only human Rd cells [82]. Although it is well known that, in addition to PCV2, outbreaks of PCVD in pigs require cofactors (e.g., PRRSV), co-infecting human cells with PCV2 and PRRSV was not yet performed. In addition to cell lines, primary human leukocytes could also be infected with PCV1, inducing severe morphological alterations in the infected cells [84], indicating that PCV1 may also be pathogenic.

When humans were screened for antibodies against PCV, in an early study, antibodies to PCV were found in 30% of samples from hospitalized patients with fever of unknown etiology [85]. These results are in striking contrast to those from another group that did not detect antibodies in serum samples from the general population and from veterinarians working with PCVD affected animals [61]. Additional studies are necessary to confirm the latter negative results.

A large “experiment” testing the susceptibility of the human population to PCV was involuntary conducted when two vaccines against rotaviral gastroenteritis from two different manufacturers were found to be contaminated with PCV1 and PCV2 [86,87,88]. Both contaminated vaccines had been used world-wide for a number of years, preventing disease and saving millions of children’s lives [89,90]. Over 10^5^ or 10^6^ particle-associated full-length PCV1 genomes were present in each dose of the contaminated vaccine [83,87,88,89], and cell culture assays in swine testis and PCV-free porcine kidney (PK-15) cells confirmed that PCV1 sequences in this vaccine represented infectious virus [86,87,88,91]. Another rotavirus vaccine contained only subgenomic PCV1 and PCV2 fragments, but no full-length PCV genomes, and cell culture assays did not amplify PCV from this vaccine [88]. When stool samples from children vaccinated with Rotarix, an oral live attenuated vaccine based on the human rotavirus RIX4414 produced by GlaxoSmithKline (London, UK), were analyzed, in 4 of 40 samples PCV1 DNA was detected [83]. PCV1 DNA was detected only soon after vaccination, indicating that viral replication did not occur in the gastrointestinal tract. Antibodies were not detected in the sera of vaccinated children, confirming that no replication of the virus had taken place. The pattern of adverse events reported in vaccinated infants with PCV1 in their stool did not differ from that observed in placebo recipients [83]. This correlated with the reports that the Rotarix vaccine in general had nearly no adverse events [89,90].

However—and this is the main question in the context of xenotransplantation—up until now it is still unknown whether PCV is zoonotic in severely immunosuppressed humans.

## 5. PCV2 and First Preclinical and Clinical Xenotransplantations

In all of the clinical xenotransplantation trials documented in Paradise et al. [92], no screening for PCV was performed in the Large White donor pigs and human recipients. Auckland Island pigs were used as source for the first clinical pig islet cell transplantation to human diabetic patients in New Zealand and Argentina [93,94,95,96]. These donor animals were free of PCV1 and PCV2, and therefore could not transmit circoviruses [94]. The sensitivity of the PCR used to detect PCV in Auckland Island pigs was estimated to be 10^6^ mg of DNA per reaction [97]. Islet cells from Auckland Island pigs were also used in a prospective pig-to-primate islet xenotransplantation study, and as expected, no PCV was transmitted [98]. In most of the reported pig-to-non-human primate transplantations, no screening for PCV was performed, with the exception of a the just mentioned trial transplanting islet cells from Auckland Island pigs into cynomolgus monkeys [98]. In addition, pig donors for islet cell transplantation into mice had also been found to be PCV-negative [99]. Recently, islet cells from Large White/Yorkshire landrace F1 pigs were transplanted into non-immunosuppressed cynomolgus monkeys, and no PCV was detected in the recipients [100]. Testing was performed based on the presence of PCV in the source herd, although the donor pigs had been vaccinated with CircoFLEX (Table 1). PCV was not tested in the monthly herd screening and in the sentinel and pancreas donor post-mortem screening, the islet cells had been encapsulated in macrobeads.

In future preclinical, as well as clinical trials, donor pigs—and, if necessary, also recipients—should be screened for the presence of circoviruses. When PCV is not found in the donor pig, no screening of the recipients needs to be performed.

## 6. Treatment and Vaccination

There is no specific treatment for pigs with PCVD. Anti-inflammatory agents and antimicrobials may help to suppress co-factors and secondary diseases associated with PCVD. All in/all out pig flow, thorough cleaning, and rigid disinfection between batches of pigs are measures that can help control the disease [103]. In the case that the donor animal is PCV-infected, it may be considered to analyze whether the xenotransplantation product (e.g., isolated islet cells) is still negative. Since PCV2 is infecting macrophages, certainly all organs are infected and it will be safer to use only negative animals, especially since no effective antiviral treatment is available. PCV2 infection is associated with an immune response including neutralising antibodies, and these coincide with a decrease in serum virus load. Cell-mediated immunity has also been shown to be necessary to control PCV2 infection (for review see [102]). PCV2 vaccines became commercially available in the summer of 2006 (Table 1) [103]. The vaccines reduced the severity and incidence rate of PCVD on many farms. Vaccination against PCV2 did not only imply a direct beneficial effect on pig productivity, but also contributed to reduction of antimicrobial use [104]. PCV2 vaccines effectively increased average daily weight gain (ADWG) and prevented diseases with a positive result for meat production. In all vaccination trials a lower virus load was registered in the vaccinated animals, however, it remains unclear whether the virus load is reduced to zero. In most reported cases, virus transmission took place despite vaccination [102,105,106,107]. In a study vaccinating 28 pigs, the virus load was not reduced to zero in any of the animals [108]. In another study, 17 of 32 vaccinated animals still showed PCV in the serum, as measured by PCR [90]. PCV2 vaccination of sows was associated with high antibody responses, but did not prevent fetal infections *in utero* or soon after birth by infectious colostrum in 29 of 100 cases [107]. When comparing four different vaccines, use of the inactivated chimeric vaccines (Fostera PCV and Circovax) resulted in significantly lower viremia compared with use of the subunit vaccines (Circoflex, Porcilis PCV), however, histopathological lesions and PCV antigens were still detected in all 80 immunized animals [109]. Successful vaccination is mainly associated with induction of neutralizing antibodies, but T cell-mediated immunity also plays a role in the reduction of the virus load and prevention of diseases as mentioned above [102,110].

Since new PCV2 variants have emerged, the question of whether or not current vaccines can protect against new PCV2 variants that may be more virulent for pigs becomes a serious concern. Although it is still unclear whether the global switch from PCV2a to PCV2b and PCV2d was associated with higher fitness of PCV2, as reported [68,69], rather than vaccine induced selection pressure, the emergence and rapid spread of new PCV2 variants provide evidence that current vaccines need to be updated.

## 7. How to Eliminate PCV

For a safe xenotransplantation, elimination programs have been proposed for porcine viruses such as HEV [17], PCMV [111], porcine lymphotropic herpesviruses, and others [112], by isolation of virus-free animals, treatment, and vaccination. Elimination programs in the case of circoviruses should be based on (i) selection of animals found non-infected using highly sensitive detection methods to avoid false-negative testing; (ii) vaccination or other strategies (see below), since treatment is not available; and (iii) isolation of virus-negative animals to prevent *de novo* infection. Elimination means elimination from the herd, elimination from a single individual is impossible, since there is no treatment presently available. The efficacy of the vaccines should be improved and new vaccines against emerging variant virus strains should be developed. Since PCV2 is easily transmitted through the placenta and since colostrum was shown to be infectious, Cesarean section, and colostrum derivation are two promising strategies to eliminate PCV [80,106,107,113]. Recent findings of PCV2 in Göttingen Minipigs [114], which were introduced into the facility by Cesarean delivery and are produced under specified pathogen-free breeding conditions that are very similar to designated pathogen-free breeding conditions [115], confirm transmission through the placenta and indicate that selection of PCV2-free animals may be difficult. However, when 384 embryos recovered from PCV2 infected pigs 10 days after inoculation were transferred to seronegative donors, no infection of the recipient pig and the piglets was observed, indicating that embryo transfer can be successfully used for the elimination of PCV2 [116].

## 8. Summary

PCV2 is a very small virus, it is stable and resistant to some disinfectants, pH, and heat, and it induces severe diseases in infected pigs. PCV2 is an immunosuppressive virus and it is still unclear whether subclinical infections of pigs may decrease the functionality of the organs required for transplantation. Vaccination against PCV2 is able to prevent diseases, but in most cases is unable to prevent the transmission of the virus. Although PCV2 infects human cells and induces a cytopathic effect in vitro, no pathogenic effects were observed when PCV was transmitted by contaminated vaccines to children. It remains unknown whether PCV may infect severely immunosuppressed individuals. In conclusion, for all of these reasons, sensitive detection methods should be used to screen for the virus and improved vaccination, Cesarean delivery, colostrum deprivation, and embryo transfer should be used to prevent transmission of the virus.

## Figures and Tables

**Table 1:** Protective vaccines against PCV2 [101,102].

Vaccine	Producer	Vaccine Based on
Circumvent PCV, Porcilis PCV, Circumvent G2 PCV	MSD/Merck Animal Health (Madison, New Jersey, United States)	PCV2a Cap protein expressed by baculovirus
Ingelvac CircoFLEX	Boehringer-Ingelheim (St. Joseph, Missouri, United States)	PCV2a Cap protein expressed by baculovirus
Fostera PCV, Suvaxyn PCV	Zoetis (Parsippany, New Jersey, United States)	Inactivated recombinant PCV1 expressing the PCV2a Cap protein (ORF2 from PCV2)
Circovac	Merial (Lyon France)	Inactivated whole PCV2a

ORF: Open reading frame.
